# Self-Concept and Achievement in Math Among Australian Primary Students: Gender and Culture Issues

**DOI:** 10.3389/fpsyg.2019.00603

**Published:** 2019-03-26

**Authors:** Feifei Han

**Affiliations:** Griffith University, Mt Gravatt, QLD, Australia

**Keywords:** gender, Australian Indigenous culture, self-concept of competence and affect, math achievement, primary school students

## Abstract

While gender stereotype on math learning and achievement is consistently reported among existing research, these studies predominantly focus on mainstream students with Western cultural backgrounds. There is a dearth of study, which investigates gender effect among Australian Indigenous students. To fill this gap, the present study adopted a multiple-indicator-multiple-indicator-cause approach to structural equation modeling to investigate effects of gender, culture (Indigenous vs. non-Indigenous), and the interaction of the two on students’ self-concept of competence and affect in math, as well as math achievement among Australian primary school students. We found gender stereotype effect not only on students’ self-perceptions of their competence in math but also their actual math performance reflected in their math achievement scores in a standard math test. Boys had higher ratings on math competence and scored more highly on math test than girls. However, the gender stereotype was not found for self-concept of affect. Instead, culture was significantly impacted on self-concept of math affect, indicating that Indigenous students had less enjoyment toward learning math compared with their non-Indigenous peers. Furthermore, significant interaction effects between gender and culture were observed on both self-concept of math competence and math affect. In practice, to enhance Indigenous students’ interest and enjoyment in math learning, educators are suggested to incorporate Indigenous students’ values, beliefs, and traditions when delivering new math knowledge.

## Introduction

Indigenous Australians are the first peoples of Australia (Craven et al., 2013) and are one of the most disadvantaged Indigenous populations in the world (Cooke et al., 2007). All Australian governments in the last two decades have acknowledged that Indigenous Australians are disadvantaged in a number of socioeconomic indicators, including education (e.g., Commonwealth of Australia, 2006). Indigenous students participate significantly less in education and have significantly higher attrition rates compared to other Australian populations. For instance, the retention rate for Indigenous students from year 7 (the first year of secondary school) to year 12 was 55%, whereas the retention rate for non-Indigenous students during this same time period was 82% (Australian Bureau of Statistics, 2012). Craven et al. (2013) have called for paying a special attention to the disparities in educational outcomes between Indigenous students and their peers and taking immediate actions to make Indigenous Australians’ full potential flourish. Thus, the first aim of the present study is to gain a thorough understanding on how Indigenous Australian students differ from non-Indigenous Australian students on self-concept and achievement in math subject in order to inform the development of effective intervention programs to help close the educational gap for Indigenous students.

Moreover, past research has revealed that there may be a gender stereotype in academic self-concept, and achievement in math (Meece et al., 2006; Yeung et al., 2012a), in particular, gender differences tend to become observable from early primary school (Eccles et al., 1993; Usher and Pajares, 2008). However, existing research regarding students’ self-concept in math predominantly focused only on the perceptions of one’s competence, neglecting the perceptions of one’s affect in math (Midgley et al., 2001; Yeung and Han, 2017). Furthermore, whether the commonly found gender stereotype in self-concept and achievement in math is also observable in Australian indigenous student population is unknown. As Australian Indigenous culture, values, and perspectives differ from the dominant Western culture, we cannot legitimately apply the gender stereotype in math self-concept and achievement from the mainstream Western culture to Australian Indigenous students. Therefore, the present study examines both the gender and culture effects and the interaction of the two, on self-concept of competence and affect, as well as achievement in math subject among Australian primary school students from Indigenous and non-Indigenous backgrounds.

## Literature Review and Research Questions

### Self-Concept and Its Impact

Positive self-beliefs are at the heart of the positive psychology movement (Marsh and Craven, 2006) and enhancing self-concept is enshrined in educational policies internationally. For example, the *Melbourne Declaration on Educational Goals for Young Australians* (MCEETYA, 2008) emphasizes that students should “have a sense of self-worth, self-awareness, and personal identity that enables them to manage their emotional, mental, spiritual, and physical wellbeing” (p. 9). Positive self-concept has been demonstrated “to impact on a wide range of critical wellbeing outcomes and serve as an influential platform for enabling full human potential” (Craven and Marsh, 2008, p. 104). Interventions specifically addressing domain-specific self-concept have been shown to result in domain-specific gains in a range of achievement outcomes (Craven and Yeung, 2008). Numerous studies have identified strong relations between self-concept and outcomes such as well-being, coursework selection, rate of school completion, adaptive academic behaviors, coping mechanisms, enhanced academic achievement, and reduced mental health problems (e.g., Marsh and Craven, 2006; Craven and Yeung, 2008). Self-concept and achievement are also known to be reciprocally related whereby they share a dynamic causal relation (Marsh and Craven, 2006). In the school context, academic self-concepts in different school subjects have been consistently demonstrated that they are not only causes for cognitive outcomes; but are also triggers of desirable psychological outcomes (Marsh and Craven, 2006; Craven and Yeung, 2008).

The structure of self-concept has been empirically demonstrated as multidimensional and domain specific (Arens et al., 2011). Marsh (1990), for example, found distinct self-concepts in a number of school subjects, including verbal, math, physical, art, music, and religion, with a general academic self-concept as an overarching construct. Traditionally, researchers either conflated the competence and affect aspects of academic concept (e.g., Jansen et al., 2014) or they have placed more emphasis on self-concept of competence over affect. Hence, academic self-concept has been consistently measured by either combining the competence and affect aspects or predominantly using competence aspect alone (Pinxten et al., 2013; Seaton et al., 2014).

However, in recent years, academic self-concept it has been empirically demonstrated that self-concept of competence (in relation to cognition) and self-concept of affect (in relation to emotion) are clearly distinguishable (e.g., Arens et al., 2011; Pinxten et al., 2013). While the competence component is concerned with the extent to which students perceive themselves to have capabilities in a specific school subject (e.g., I am good at math.), the affect component is about the extent to which an individual enjoys participating in a subject (e.g., I like math.). Thus, we will examine both the competence and affect aspects of self-concept in math.

### Gender and Culture Issues in Self-Concept and Achievement

Gender differences may be observable as early as in elementary school when self-beliefs and perceptions begin to form (Eccles et al., 1993). Past self-concept research focusing only on competence has indicated that in general boys tend to have higher competence beliefs than girls (Midgley et al., 2001). Boys sometimes overestimate their competence whereas girls tend to underestimate theirs (Metallidou and Vlachou, 2007), even though such self-perceptions of abilities may not match their real ability (Yeung et al., 2012a,b). However, boys and girls may exhibit very different levels of competence beliefs in different curriculum areas. Research has consistently reported that boys tend to have higher perceptions of competence in math and science-related subjects (Marsh and Yeung, 1998; Klapp Lekholm and Cliffordson, 2009), whereas girls show higher self-concepts in language and verbal-related subjects (Marsh, 1993; Kurtz-Costes et al., 2008).

Gender has also been found to interact with other factors, including students’ ability and cultural backgrounds (Dai, 2001; Chiu and Klassen, 2010). For instance, Dai (2001) observed that for average-ability students, girls reported a higher verbal self-concept and lower math self-concept than boys. However, for gifted students, girls were found to have a comparable math self-concept to boys. Chiu and Klassen (2010) found that for students from a culture which tolerates more uncertainty, math self-concept had a stronger relation with math achievement for boys than for girls. Extending a focus on self-concept of competence to affect beliefs, Yeung et al. (2012a,b) found an interaction effect between culture and gender—the competence and affect differences between Asian and Anglo Australian students were more pronounced for boys than for girls.

With regard to academic achievement, interaction effects between culture and gender have also been demonstrated. Lai (2010), for instance, showed that Chinese girls performed better than boys in both primary and middle schools. But for American students, this pattern was not consistent. American girls achieved better than boys in elementary school, but boys gradually caught up in math and science in middle schools.

### The Current Study and Research Questions

Although the above studies demonstrated that gender effects on academic self-concept and achievement may be partly influenced by culture, there is a lack of research on how gender effect may be interacted with Australian Indigenous culture. In this investigation, we will test gender, Indigenous culture, and the interaction of the two on the self-concept of math competence and affect, as well as math achievement among Australian primary school students.

Three research questions were addressed in the study:

1. Do boys and girls differ in the self-concept of math competence, affect, and math achievement?
2. Do Indigenous and non-Indigenous students differ in the self-concept of math competence, affect, and math achievement?
3. Are there interaction effects between gender and Indigenous culture on the self-concept of math competence, affect, and math achievement?

## Materials and Methods

### Participants

The study was conducted with 566 boys (44.6%) and 702 girls (55.4%), who studied in urban and rural areas of Australian primary schools. The students were from grade 3 (average age around 10) to grade 6 (average age around 13). Among them, 496 (39.1%) self-identified as Indigenous background in the demographic information, whereas 772 (60.9%) were self-identified as non-Indigenous students.

### Materials

The materials used for data collection were a self-report questionnaire and students’ scores in a standard math achievement test. The questionnaire started with a section on demographic information including age, gender, grade, and cultural background followed by items on self-concept of math competence and affect, which are explained in detail below.

### Math Competence and Math Affect Scales

To measure students’ self-concept of math competence, we used the four positive items from Marsh (1992) Self Description Questionnaire I (SDQI). These items are: “I learn things quickly in math,” “Work in math is easy for me,” “I get good marks in math,” and “I am good at math.” The items which evaluated students’ self-perceptions of affect toward math were also from SDQI, including: “I like math,” “I am interested in math,” “I look forward to math,” and “I enjoy doing work in math.” We excluded the two negatively worded items from the original math competence and affect scales because past research showed that incorporating negative items resulted in negative item bias and reduced the reliability of scales (Marsh, 1986; Arens et al., 2011). The reason for using SDQI is that it is the most widely used instrument for measuring self-concept of students from diverse cultures, including Australian Indigenous students (e.g., Worrell et al., 2008; Bodkin-Andrews et al., 2010). All the items were on a 5-point scale with 1 representing strongly disagree and 5 indicating strongly agree.

### Math Achievement Scores

The math achievement scores were obtained using a state-wide standardized test organized by Department of Education and Training. The test lasted about 20 min and had different items for different grades (see the Appendix for the sample items).

### Data Collection Procedure

The data collection strictly followed the ethics requirements. Before administering the study, the written consent from the participants and their parents for voluntary participation was obtained. The effort was made to ensure the anonymity of the participants by assigning a code to each participating student. The questionnaire was administered in groups by research assistants, who read each item aloud to the students to minimize potential problems arising from reading difficulties.

### Data Analysis

We started data analysis by constructing a CFA model with four items of self-concept of math competence, four items of self-concept of math affect, and the math achievement scores using Mplus version 7. Because the math achievement scores were a single-item indicator, the measurement error of scores was fixed with a perfect reliability estimate (Marsh and Yeung, 1997).

The criteria for the evaluation of CFA models followed the general procedures proposed by Kline (2005) and Jöreskog and Sörbom (2005). We considered four fit statistics as our primary indicators of model fit, namely the Comparative Fit Index (*CFI*, Bentler, 1990), the Tucker-Lewis Index (*TLI*, Tucker and Lewis, 1973), the root mean squared residual (*SRMR*, Bentler, 1995), and the root mean square error of approximation (*RMSEA*, Browne and Cudeck, 1993). The values of *TLI* and *CFI* higher than 0.950, *SRMR* less than 0.080, and *RMSEA* below 0.060 are generally considered as good fit between the hypothesized model and the observed data (Bentler, 1990; Browne and Cudeck, 1993; Hu and Bentler, 1999). Besides these fit statistics, support for the fit of CFA models also requires: (1) acceptable reliability for each scale (i.e., *α* = 0.700 or above); (2) factor loadings of items above 0.300 on the corresponding scales, and (3) appropriate correlations among the latent factors to ensure that they are distinguishable from each other (*r*s below 0.900).

When the CFA model was established (model 1), we then conducted a series of measurement invariance tests to determine if the CFA model was equivalent across female and male students (i.e., gender), and across Indigenous and non-Indigenous students (i.e., culture). The invariance tests involve evaluating various levels of restricted models and proceed in a stepwise manner from loosest to tightest. Therefore, the invariance models are nested because the imposed constraints are progressively added (Brown, 2006; Byrne, 2016). We first constructed three models (models 2A–2C) to examine measurement invariance by gender. We followed Brown’s recommended procedure for performing invariance tests by starting from a configural CFA (model 2A), which is the least restricted model, tests whether the factor structures are identical across groups. Following the configural model, we tested whether factor loadings were equal in the metric model (model 2B). We then constrained intercepts to be equal, referred to as the scalar model (model 2C). Similarly, the next three successive models (models 3A–3C) were constructed to test whether measurement equivalence could be attained across the two cultural groups. Model 3A was a configural CFA model that examines whether the factor structures are identical across Indigenous and non-Indigenous students. Model 3B was the metric model, in which all the factor loadings were constrained to be equal across the two cultural groups of students, and model 3C was the scalar model, in which the equal constraints were put on both the factor loadings and the intercepts.

To examine the effect of gender, culture, and the interaction of the two on students’ self-concept of math competence, math affect, and math achievement, we adopted a multiple-indicator-multiple-indicator-cause (MIMIC) approach to structural equation modeling (Aiken and West, 1991; Jöreskog and Sörbom, 2005; Marsh et al., 2005). The advantage of a MIMIC model is that measurement errors of latent variables are corrected (Marsh et al., 2006; Yeung et al., 2012b). The MIMIC model (model 4) examined the multiple causes of the three discrete grouping variables (1) gender (1 = boy, 2 = girl), (2) culture (1 = Indigenous, 2 = non-Indigenous), and (3) gender × culture interaction to students’ self-concept of math competence, math affect, and math achievement.

## Results

### CFA of Model 1

The CFA of model 1 produced a good fit to the data: *χ*^2^ (25) = 188.039, *CFI* = 0.980, *TLI* = 0.970, *SRMR* = 0.019, *RMSEA* = 0.072 (Table 1). All factor loadings of items on their corresponding scales were above 0.750, and both self-concept of math competence and math affect scales were highly reliable (0.891 and 0.924, respectively). The correlations between math competence, affect, and math achievement scores are presented in Table 2, which shows that all the correlations are significant and positive—math competence and math affect: *r* = 0.815, *p* < 0.010; math competence and math achievement: *r* = 0.105, *p* < 0.010; math affect and math achievement: *r* = 0.057, *p* < 0.050. The results of correlations showed that the relation between self-concept of math competence and math affect was substantial, whereas both the relations between self-perceptions of math competence and achievement and between perceptions of math affect and achievement were weak.

**Table 1:** Goodness of fit of models.

Models	^χ 2^	*df*	*CFI*	*TLI*	*SRMR*	*RMSEA*
Model 1 (CFA of latent factors)	188.039	25	0.980	0.970	0.019	0.072
Model 2A (gender invariance-configural)	245.129	50	0.977	0.967	0.022	0.078
Model 2B (gender invariance-metric)	248.364	56	0.977	0.971	0.024	0.074
Model 2C (gender invariance-scalar)	250.971	62	0.978	0.974	0.025	0.069
Model 3A (culture invariance-configural)	239.216	50	0.978	0.968	0.021	0.077
Model 3B (culture invariance-metric)	244.244	56	0.978	0.971	0.025	0.073
Model 3C (culture invariance-scalar)	254.357	62	0.977	0.974	0.027	0.070
Model 4 (MIMIC model)	214.924	43	0.980	0.970	0.017	0.056

CFI = comparative fit index; TLI = Tucker-Lewis index; SRMR = standardized root mean square residual.

**Table 2:** Correlation between math competence, math affect, and math achievement.

Variables	Math affect	Math achievement
Math competence	0.815**	0.105**
Math affect	—	0.057*

* p < 0.050;

** p < 0.010.

### Factorial Invariance Across Groups

The results of a series of invariance tests are summarized in Table 1. Table 1 shows that the baseline model (model 2A) resulted in a good fit: *χ*^2^ (50) = 245.129, *CFI* = 0.977, *TLI* = 0.967, *SRMR* = 0.022, *RMSEA* = 0.078. Both model 2B: *χ*^2^ (56) = 248.364, *CFI* = 0.977, *TLI* = 0.971, *SRMR* = 0.024, *RMSEA* = 0.074; and model 2C: *χ*^2^ (62) = 250.971, *CFI* = 0.978, *TLI* = 0.974, *SRMR* = 0.025, *RMSEA* = 0.069, produced similar fits to model 2A, providing evidence for the equivalence of the measurement structure across the boy and the girl groups (Cheung and Rensvold, 2002). Following the same procedure, for the invariance of culture groups, we found that the baseline model (model 3A) yielded an appropriate fit: *χ*^2^ (50) = 239.216, *CFI* = 0.978, *TLI* = 0.968, *SRMR* = 0.021, *RMSEA* = 0.077. Across the two culture groups, the fit statistics of model 3B: *χ*^2^ (56) = 244.244, *CFI* = 0.978, *TLI* = 0.971, *SRMR* = 0.025, *RMSEA* = 0.073; and 3C: *χ*^2^ (62) = 254.357, *CFI* = 0.977, *TLI* = 0.974, *SRMR* = 0.027, *RMSEA* = 0.070, were comparable to those of model 3A, supporting the factorial invariance across Indigenous and non-Indigenous groups.

### Paths of the MIMIC Model

**Figure 1:**
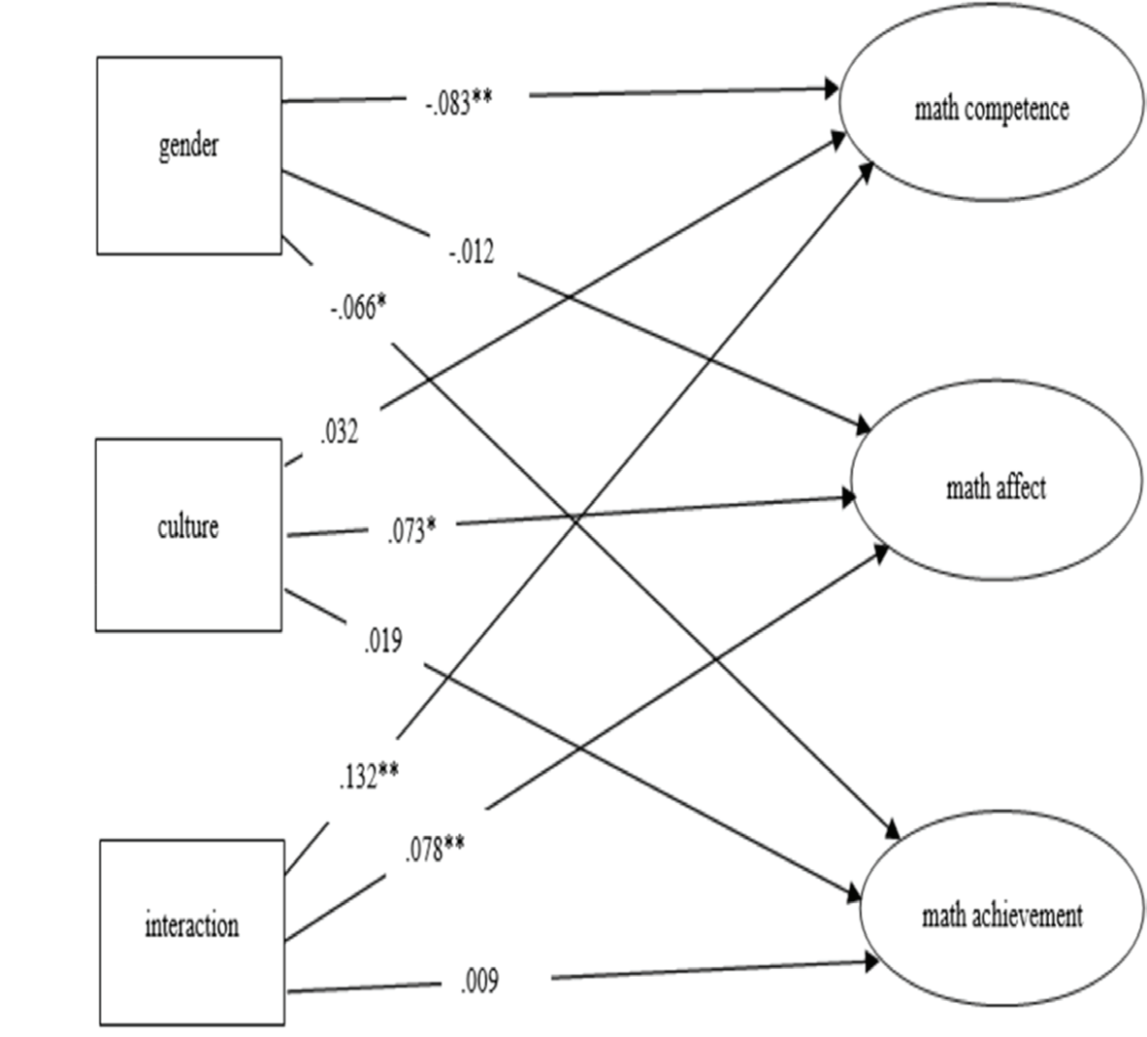
Paths of the MIMIC model. ^*^p < 0.010; ^**^p < 0.050.

The invariance of measurement across groups allowed us to proceed with MIMIC model (model 4), which displayed a good fit to the data: *χ*^2^ (43) = 214.924, *TLI* = 0.980, *CFI* = 0.970, *SRMR* = 0.017, *RMSEA* = 0.056. The factor loadings and paths of model 4 are displayed in Table 3 and the MIMIC model is also displayed in Figure 1. The descriptive statistics of self-concept of math competence, math affect, and math achievement by gender and culture are presented in Table 4.

**Table 3:** Solution of model 4.

Variables	Math competence	Math affect	Math achievement
Factor loadings			
Item 1	0.753**	0.818**	1.000
Item 2	0.834**	0.866**	—
Item 3	0.830**	0.897**	—
Item 4	0.853**	0.891**	—
Uniqueness			
Item 1	0.433**	0.331**	0.000
Item 2	0.304**	0.251**	—
Item 3	0.311**	0.195**	—
Item 4	0.272**	0.207**	—
Gender	−0.083**	−0.012	−0.066*
Culture	0.032	0.073*	0.019
Interaction	0.132**	0.078**	0.009

* p < 0.050;

** p < 0.010.

**Table 4:** Descriptive statistics by gender and culture.

Variables	Indigenous boys (*N* = 215)	Non-Indigenous boys (*N* = 351)	Indigenous girls (*N* = 218)	Non-Indigenous girls (*N* = 421)
	*M* (*SD*)	*M* (*SD*)	*M* (*SD*)	*M* (*SD*)
Math competence	3.992 (1.200)	4.154 (1.064)	3.833 (1.226)	3.935 (1.063)
Math affect	3.834 (1.393)	4.067 (1.223)	3.803 (1.350)	4.010 (1.193)
Math achievement	51.737 (10.580)	56.506 (9.860)	50.591 (8.915)	56.592 (9.970)

From Table 3, we can see that the main effect of gender was significant and negative for self-concept of math competence (*β* = −0.083, *p* < 0.010) and math achievement (*β* = −0.066, *p* < 0.050). The significant and negative path coefficients suggested that boys not only had higher ratings on their perceptions of abilities in math compared with girls (boys: *M* = 4.092, *SD* = 1.119; girls: *M* = 3.894, *SD* = 1.131), they also obtained higher scores in the math achievement test (boys: *M* = 54.695, *SD* = 9.998; girls: *M* = 54.190, *SD* = 10.175). The coefficients of the paths from culture were only significant for self-concept of math affect (*β* = 0.073, *p* < 0.050). The positive path suggested that Indigenous students had less enjoyment toward learning math compared with their non-Indigenous peers (Indigenous students: *M* = 3.817, *SD* = 1.367; non-Indigenous students: *M* = 4.036, *SD* = 1.208).

Statistically significant interaction effects between gender and culture were also found on both self-concept of math competence (*β* = 0.132, *p* < 0.010) and math affect (*β* = 0.078, *p* < 0.010). For self-concept of math competence, while non-Indigenous boys (*M* = 4.154, *SD* = 1.064) had higher ratings than non-Indigenous girls (*M* = 3.935, *SD* = 1.063), there was no significant difference between Indigenous boys (*M* = 3.992, *SD* = 1.200) and Indigenous girls (*M* = 3.833, *SD* = 1.226). For self-concept of math affect, non-Indigenous boys (*M* = 4.067, *SD* = 1.223) had higher ratings than Indigenous girls (*M* = 3.803, *SD* = 1.350), whereas there was no significant difference between non-Indigenous girls (*M* = 4.010, *SD* = 1.193) and Indigenous boys (*M* = 3.834, *SD* = 1.393). *Post hoc* power analyses indicated that with the sample size of the study, the power to detect obtained effects at the 0.050 level was 0.999 in prediction of math competence, was 0.874 in prediction of math affect, and was 0.867 in prediction of math achievement.

The present study investigated the effect of gender, culture, and the interaction of the two on Australian primary school students’ self-concept of math competence and affect, as well as their math achievement. Two separate sets of measurement invariance tests on examination of invariant factor structure, factor loadings, and intercepts across both gender and culture demonstrated that female and male students, Indigenous and non-Indigenous students shared the same interpretation of the items with regard to their self-evaluation and perceptions of their capabilities and liking toward the math subject. Because of the identical pattern of factor-indicator relationships, factor loadings, and intercepts, the factor scores from the four subgroups of the sample (i.e., Indigenous boys, non-Indigenous boys, Indigenous girls, and non-Indigenous girls) can be legitimately compared. The examination of the measurement invariance ensured the potential use of the PBLEQ in various academic disciplines in higher education. The MIMIC approach found that gender differences were not only in students’ perceived capabilities in the processes of learning math (self-concept of math competence) but also evident in their actual abilities in solving math problems (math achievement). Consistent with the past findings on the gender stereotype, our study also showed that girls’ self-perceptions and confidence in evaluating their competence in math was lower than boys (e.g., Eccles et al., 1993; Marsh, 1993; Marsh and Yeung, 1998; Kurtz-Costes et al., 2008; Klapp Lekholm and Cliffordson, 2009). However, when taking the culture effect into consideration, we found such difference only existed among the non-Indigenous students. Such result may suggest that the general finding of the gender stereotype is only applicable among the mainstream students from Western cultural background given the fact that the majority of past studies did not investigate Indigenous population.

By separating the cognitive (competence) and affective (affect) aspects of math self-concept, our findings further extended the examination of gender effect of math competence to math affect. We observed that different from gender stereotype in self-perceptions of math competence, no gender effect was found on students’ self-perceptions of liking and enjoyment of studying math subject. This means that boys and girls had similar ratings on their enjoyment of learning math. Instead, gender only had a significant impact on self-concept of math affect when it interacted with cultural backgrounds. Generally speaking, Indigenous students reported less enjoyment in learning math compared with their non-Indigenous counterparts. However, there was no such difference between Indigenous boys and non-Indigenous girls. Out of the expectation, there was no achievement gap between Indigenous and non-Indigenous students on their math achievement scores. Rather, students’ performance on the math achievement test was consistent with how they self-evaluated their competence, as the results showed a gender stereotype of math achievement among Australian primary school students regardless whether they were from an Indigenous or a non-Indigenous background.

### Implications for Educational Practice

In educational practice in Australian primary school contexts, teachers may need to make some efforts in boosting Australian female primary school students’ beliefs of their competence in math subject. Based on the known reciprocal effects between self-concept and achievement (Marsh and Craven, 2006), students are likely to improve their math performance through interventions that focus on boosting their confidence in math competence. Furthermore, educators need to pay special attention to Indigenous students’ lower interest and enjoyment in math learning. Past research has shown that when teaching is designed to incorporate Indigenous students’ values, beliefs, and traditions, and when new knowledge is delivered in a way that is culturally appropriate to Indigenous students, the learning tends to be more effective (e.g., Gitari, 2006; Yunkaporta, 2009). Thus, to nurture Indigenous students’ interests in math learning, teachers may consider using some materials which are able to foster relevance to Indigenous students’ culture.

## Data Availability

The datasets generated for this study are available on request to the corresponding author.

## Ethics Statement

This study was carried out in accordance with the recommendations of “National Statement on Ethical Conduct in Human Research, the Human Research Ethics Committee of Western Sydney University” with written informed consent from all subjects and their parents. All subjects and their parents gave written informed consent in accordance with the Declaration of Helsinki. The protocol was approved by “the Human Research Ethics Committee of Western Sydney University.”

## Author Contributions

FH contributed to the conception of the work and to the acquisition, analysis, and interpretation of the data; drafted the work and revised it critically for important intellectual content; approved the final version of the paper to be published, and agreed to be accountable for all aspects of the work in ensuring that questions related to the accuracy or integrity of any part of the work are appropriately investigated and resolved.

### Conflict of Interest Statement

The author declares that the research was conducted in the absence of any commercial or financial relationships that could be construed as a potential conflict of interest.
